# A Hybrid Opinion Formation and Polarization Model

**DOI:** 10.3390/e24111692

**Published:** 2022-11-19

**Authors:** Baizhong Yang, Quan Yu, Yi Fan

**Affiliations:** 1 School of Mathematics and Statistic, Guizhou University, Guiyang 550025, China; 2 School of Mathematics and Statistic, Qiannan Normal University for Nationalities, Duyun 558000, China; 3 Key Laboratory of Complex Systems and Intelligent Optimization of Guizhou Province, Duyun 558000, China

**Keywords:** opinion dynamics, opinion formation, opinion polarization

## Abstract

The last decade has witnessed a great number of opinion formation models that depict the evolution of opinions within a social group and make predictions about the evolution process. In the traditional formulation of opinion evolution such as the DeGroot model, an agent’s opinion is represented as a real number and updated by taking a weighted average of its neighbour’s opinions. In this paper, we adopt a hybrid representation of opinions that integrate both the discrete and continuous nature of an opinion. Basically, an agent has a ‘Yes’, ‘Neutral’ or ‘No’ opinion on some issues of interest and associates with its Yes opinion a support degree which captures how strongly it supports the opinion. With such a rich representation, not only can we study the evolution of opinion but also that of support degree. After all, an agent’s opinion can stay the same but become more or less supportive of it. Changes in the support degree are progressive in nature and only a sufficient accumulation of such a progressive change will result in a change of opinion say from Yes to No. Hence, in our formulation, after an agent interacts with another, its support degree is either strengthened or weakened by a predefined amount and a change of opinion may occur as a consequence of such progressive changes. We carry out simulations to evaluate the impacts of key model parameters including (1) the number of agents, (2) the distribution of initial support degrees and (3) the amount of change of support degree changes in a single interaction. Last but not least, we present several extensions to the hybrid and progressive model which lead to opinion polarization.

## 1. Introduction

In social life, opinions and beliefs significantly affect human choices and also drive their actions [1]. Therefore, it is important to understand opinion dynamics, i.e., the evolution process of opinion spreading and forming in social networks. Opinion dynamics can be applied in various aspects [2,3,4,5,6,7,8]. For example, in political elections, Bravomarquez et al. [9] conducted an empirical study on the opinion time series in the 2008 American election by using Twitter data. In market research, Castro et al. [10] proposed a recommendation system based on opinion dynamics to help users choose the right product or service in a scenario of excessive information. In research on transportation, Hashemi et al. [11] proposed an opinion dynamics method to improve the reliability of the speed estimator. In other fields, Noah et al. [12] studied the evolution of the American people’s opinions on a series of issues related to the Iraq war. Carmela et al. [13] explained the mechanism of consensus reached by 178 countries in the 2015 Paris Climate Change Agreement, etc. In this way, researchers have deepened their understanding of the formation and evolution of opinions and aroused interest from other fields.

Models in opinion dynamics usually include three elements: expression formats of opinions, fusion rules and dynamic environments of opinions. In particular, the agents in the group express initial opinions through a special expression format. According to fusion rules, the opinions of the agents are updated repeatedly. Finally, the opinions of all agents form a stable state: consensus, polarization or fragmentation. According to whether the opinion values are discrete or not, the opinion dynamics can be divided into two categories: (1) discrete opinion models, e.g., the Ising model [14,15,16,17,18,19], the Sznajd model [20,21,22], the Voter model [23,24,25,26,27,28], the majority-vote model [29,30,31,32,33], and (2) continuous opinion models, e.g., the Deffuant–Weisbuch (DW) model [34,35,36,37] and the Hegselmann–Krause (HK) model [38,39,40,41,42]. The former type usually describes situations in which agents have a finite number of opinions. As for the latter type, the DW model updates asynchronously and allows two agents to interact with each other if their opinions are close to some extent, while the HK model updates synchronously and allows a crowd of agents to do so simultaneously if their opinions are somewhat similar. In addition, both the DW and the HK models rely on the idea of repeated averaging under a confidence threshold. Considering these works, we believed that both discrete and continuous models have disadvantages and thus we will propose a hybrid model where opinions are discrete (support, oppose, feel neutral) while support degrees are continuous (lying in the range of [0,1] with 0 meaning absolutely oppose and 1 meaning absolutely support). First discrete opinions are tailored for some situations, one of which may be voting for some representatives in congress or parliament. Second continuous support degrees reflect delicate feelings and emotions, which are natural in real life.

Most studies on continuous opinion dynamics take a weighted average of agent opinions in any single interaction [43,44,45]. However, in reality, when an agent is exposed to its same opinion, its confidence in this opinion will be strengthened. Moreover, when two agents meet with different opinions, they may not be able to make their opinions the same immediately. In fact, there are many versions of opinion dynamics models that take into account the “support” or “conviction” of an agent. For example, Roy et al. [46] studied this public and private opinion dynamics and the critical behaviour of the consensus-forming transitions using a kinetic exchange model; Szurlej et al. [47] studied the binary q-voter model with generalized anticonformity on random Erdős–Rényi graphs; Lallouache et al. [48] proposed a minimal multiagent model for the collective dynamics of opinion formation in society by modifying kinetic exchange dynamics studied in the context of income, money or wealth distributions in a society; Scheufele et al. [49] studied how the opinion climate affects participatory behaviour with or without public expression of opinion. Yet none of these studies allows opinions to be strengthened when like-minded agents meet. Therefore, in this paper, we will propose a novel model called progressive opinion evolution (POE) which exploits a slow and continuous accumulation updating strategy to deal with the drawbacks above. Based on this model, we will mainly discuss how agents interact and update their opinions.

To be specific, we proposed an updating rule for agents’ support degrees, i.e., how strongly they support an opinion, and thus constructed a mathematical model accordingly. Moreover, we conducted simulations to test parameter sensitivity on evolution processes. Our main contributions are summarized as follows: (1) a framework for opinion formation through progressive opinion change; (2) three mechanisms for opinion polarization.

The remainder of this paper is organized as follows. Section 2 presents some necessary preliminaries. Section 3 describes our progressive evolution model. Section 4 presents empirical evaluations of the effects of different parameters on opinion evolution. Section 5 discusses polarization mechanisms as well as related simulations. Finally, Section 6 concludes this paper and discusses future works.

## 2. Preliminaries

In the simulations part, we discuss groups of agents whose support degrees about an opinion follow certain distributions, so we introduce notations concerning some probability distributions here. We use X∼U[a,b] to denote that *X* follows a uniform distribution over [a,b]. Moreover, we use $X∼N(\mu ,\sigma^{2})$ to denote that *X* follows a normal distribution with μ and $\sigma^{2}$ as its mean and variance, respectively. On the other hand, we use X∼beta(α,β) to denote that *X* follows a beta distribution, where α>0 and β>0, respectively. Moreover, we sometimes talk about a range of values, so for simplicity, we use E(a,t,b) to denote a set of numbers that begin with *a* and do not exceed *b* with *t* as a single step, i.e., E(a,t,b)={a+k·t|a+k·t≤b,k∈Z,k≥0}.

## 3. The Proposed Model

Consider a set of agents, $A=\{a_{1},\cdots ,a_{N}\}$, and a discrete-time stamp t∈{0,⋯,∞} at which opinions update. To demonstrate how strongly an agent supports an opinion, we first introduce the definitions of *support degree* and *opinion* as below.

**Definition 1.**  *Given an agent $a_{i}$ and a time stamp t, we define its support degree $s_{i}\left(t\right)$ as a function with a range [0,1]. Moreover, we define opinions as*

(1) $$x_{i}\left(t\right)=\left\{\begin{array}{cc} 1, & \;\mathrm{if}\;s_{i}\left(t\right)>0.5;\\ 0, & \;\mathrm{if}\;s_{i}\left(t\right)=0.5;\\ -1, & \;\mathrm{if}\;s_{i}\left(t\right)<0.5. \end{array}\right.$$

In our setting, if an agent’s support degree is greater than (resp. smaller than) 0.5, we say that it supports (opposes) an issue. Otherwise, we say that he remains neutral about an issue. In what follows, we use 0≤δ≤1 to denote support degree change (SDC), the increase or decrease of an agent’s support degree. The larger δ is, the more significant an agent’s support degree update.

Below, we present the definition of *support degree profile* which describes the support degree of all agents.

**Definition 2.**  *Given a time stamp t, the support degree profile (SDP) at time t, denoted by S(t), is defined as $\langle s_{1}\left(t\right),\cdots ,s_{N}\left(t\right)\rangle$, which is a vector of support degrees of all agents.*

Below, we define special cases which will be useful for introducing what we mean by consensus.

**Definition 3.**  *If $s_{i}\left(t\right)>0.5$ (resp. $s_{i}\left(t\right)<0.5$, $s_{i}\left(t\right)=0.5$) for all 1≤i≤N, we say that S(t) is a positive (resp. negative, neutral) SDP.*

In this paper, we will only be interested in cases where the initial SDP is neither positive nor negative nor neutral. Next, we define a special case that will be useful in discussing polarization.

**Definition 4.**  *If ∃1≤h≠l≤N s.t.*

*1*  *$s_{h}\left(t\right)<s_{l}\left(t\right)$;*
*2.*  *∀1≤i≤N, $s_{i}\left(t\right)\notin \left(s_{h}\left(t\right),s_{l}\left(t\right)\right)$;*
*3.*  *∃j,k s.t. $s_{j}\left(t\right)\leq s_{h}\left(t\right)$ and $s_{k}\left(t\right)\geq s_{l}\left(t\right)$,*
*4.*  *and $s_{l}\left(t\right)-s_{h}\left(t\right)>0.5$;*

*then we say that S(t) is a τ*-gap* SDP, where $\tau =s_{l}\left(t\right)-s_{h}\left(t\right)$.*

Now, we show the intuition of the notion of a τ-gap SDP. (1) Item 2 implies that no agents have support degree between that of $s_{h}\left(t\right)$ and $s_{l}\left(t\right)$; i.e., the support degrees of $a_{h}$ and $a_{l}$ must be adjacent to each other in the sorted form of S(t). (2) Item 3 indicates that there must exist agents whose support degrees lie at both sides of that of $a_{h}$ and $a_{l}$ in the sorted form of S(t). (3) Item 4 ensures that our definition is well-defined as is stated in Proposition 1.

**Proposition 1.**  *At some certain time stamp, if an SDP is τ-gap, then it cannot be $\tau^{\prime }$-gap where $\tau^{\prime }\neq \tau$.*

**Proof.**  (by contradiction) Assume that there exists a profile S(t) that is both τ-gap and $\tau^{\prime }$-gap where $\tau \neq \tau^{\prime }$. According to Definition 4, τ>0.5 and $\tau^{\prime }>0.5$. Since S(t) is τ-gap, there must exist an interval of length τ where no agents have support degrees. Similarly, there must exist another interval of length $\tau^{\prime }$ where no agents have support degrees. In this sense, the intervals above are disjoint. Therefore, the length of their union is $\tau +\tau^{\prime }>1$ that exceeds the length of the interval (0,1) which is 1.The contradiction falsifies our assumption and thus confirms the validity of our proposition.    □

Notice that given an SDP S(t), if $s_{i}\left(t\right)\in \left\{0,1\right\}$ for 1≤i≤N, then it is a one-gap profile. Below, we have a proposition that asserts that in a τ-gap SDP there cannot be any neutral agents and there must exist agents with opposite opinions.

**Proposition 2.**  *If S(t) is a τ-gap SDP for some τ, then*

*1.*  *∃1≤i≤N s.t. $s_{i}\left(t\right)=0.5$;*
*2.*  *∃1≤j≠k≤N s.t. $s_{i}\left(t\right)>0.5$ and $s_{j}\left(t\right)<0.5$.*

Based on the proposition above, we are ready to understand the notion of most swinging agents as well as their implications.

**Definition 5.**  *If S(t) is a τ-gap SDP, $s_{h}\left(t\right)=\max_{s_{i}\left(t\right)<0.5}s_{i}\left(t\right)$ and $s_{l}\left(t\right)=\min_{s_{j}\left(t\right)>0.5}s_{j}\left(t\right)$, then we say that $a_{h}$ (resp. $a_{l}$) is a/the most swinging agent involved in S(t) that opposes (resp. supports) an issue.*

In this sense, considering all agents, the opinions of $a_{h}$ and $a_{l}$ are the closest to neutral. To some extent, they are the most able to be persuaded and then converted. Hence, it is reasonable to adopt their support degrees to measure the difference between the supporting sub-group and the opposing sub-group. The larger the support degree difference between $a_{h}$ and $a_{l}$, the more polarized the two sub-groups. This leads to the proposition below, in which the rationality of Definition 4 is shown.

**Proposition 3.**  *Suppose S(t) is a τ-gap SDP, $a_{h}$ and $a_{l}$ are a/the most swinging agent involved in S(t) that opposes and supports an issue, respectively, then $s_{l}\left(t\right)-s_{h}\left(t\right)=\tau$.*

Since we studied opinion dynamics empirically, we introduce definitions below which give exact meanings of observations. Below, we present what we mean by observing a process of opinion evolution that follows a certain model.

**Definition 6.**  *If $R={\langle S\left(0\right),\cdots ,S\left(T\right)\rangle }_{M}$ is a sequence of observed profiles that follows M, where T is a specified time stamp, then we say that R is an observed process of opinion evolution that follows M and T is the cutoff. Or we say that R is an observed evolution process for short if understood from the context.*

Below, we show the meaning of observing consensus or polarization of length $\left(T-t^{*}\right)$.

**Definition 7.**  *Suppose that $R={\langle S\left(0\right),S\left(1\right),\cdots ,S\left(T\right)\rangle }_{M}$ is an observed evolution process.*

*1.*  *if there exists $1\leq t^{*}\leq T$, s.t. S(t) is a positive (resp. negative, neutral) SDP for $t^{*}\leq t\leq T$, but $S(t^{*}-1)$ is not, then we say that R is observed to form a consensus of length $\left(T-t^{*}\right)$.*
*2.*  *if there exists $1\leq t^{*}\leq T$, and $\tau_{0}$, s.t. S(t) is a τ-gap SDP for $t^{*}\leq t\leq T$ with $\tau \geq \tau_{0}$ but $S(t^{*}-1)$ is not, then we say that R is observed to form a $\tau_{0}$-polarization of length $\left(T-t^{*}\right)$.*

In our setting, at each time stamp, exactly two agents meet each other, which is similar to the DW model [34]. According to their support degrees before the meeting, there are six combinations of support degrees that need to be considered (as is shown by ①∼⑥ in Table 1 (Since this table is symmetric, the below left part is ignored)):

1. Both are greater than 0.5;
2. One is greater than 0.5 while the other is less than 0.5;
3. One is greater than 0.5 while the other is equal;
4. Both are less than 0.5;
5. One is less than 0.5 while the other is equal;
6. Both equal 0.5.

**Table 1:** Cases that are considered.

$a_{i}$	$a_{j}$	>0.5	<0.5	=0.5
>0.5		①	②	③
<0.5		-	④	⑤
=0.5		-	-	⑥

Both are greater than 0.5 (See ①). One is greater than 0.5 while the other is less than 0.5 (See ②). One is greater than 0.5 while the other is equal(See ③). Both are less than 0.5 (See ④). One is less than 0.5 while the other is equal (See ⑤). Both equal 0.5 (See ⑥).

Then our update rules will be defined based on the cases above. For example, when two agents with the same opinion meet each other, both their support degrees will be increased or decreased, depending on whether they support or oppose an issue.

**Example 1.**  *Suppose that two agents both have the same support degree change δ,*

*1.*  *(both positive) if their previous support degrees are 0.6 and 0.7, then their degrees will increase to 0.6+δ and 0.7+δ, respectively;*
*2.*  *(both negative) if their previous support degrees are 0.2 and 0.3, then their degrees will decrease to 0.2−δ, and 0.3−δ, respectively.*

When two agents with opposite opinions meet each other, their support degrees will be increased or decreased and get close to each other.

**Example 2.**  *Suppose that two agents both have the same support degree change δ, if their previous support degrees are 0.4 and 0.5, then their support degrees will come close to being 0.4+δ and 0.5−δ, respectively.*

In addition, if an agent feels neutral about an issue, its opinion will be dragged and thus changed by any other one that supports or opposes this issue.

Our progressive opinion evolution (POE) model adopts asynchronous update rules, i.e., at every time stamp, two or more agents are randomly selected to communicate with each other and then update their support degrees. Yet in our models, we only allow interactions between exactly two agents. When two agents, namely $a_{i}$ and $a_{j}$, meet each other at time *t*, their support degree updates can be described as follows, and are divided into several cases depending mainly on whether they have the same or different opinions.

1. The most trivial case is that both agents are neutral; then no updates are needed, so the rule, in this case, is as below.
  (2) $$\left\{\begin{array}{c} s_{i}\left(t+1\right)=s_{i}\left(t\right),\\ s_{j}\left(t+1\right)=s_{j}\left(t\right) \end{array}\right.$$
2. If both agents are positive (resp. negative) at time *t*, their confidence will be strengthened and thus their support degrees will be increased (resp. decreased) by δ, as shown in Equations (3) and (4).
  (3) $$\left\{\begin{array}{c} s_{i}\left(t+1\right)=s_{i}\left(t\right)+\delta ,\\ s_{j}\left(t+1\right)=s_{j}\left(t\right)+\delta \end{array}\right.$$
  (4) $$\left\{\begin{array}{c} s_{i}\left(t+1\right)=s_{i}\left(t\right)-\delta ,\\ s_{j}\left(t+1\right)=s_{j}\left(t\right)-\delta \end{array}\right.$$
3. If two agents with opposite opinions meet each other, their confidence in previous opinions will be weakened, i.e., one support degree will be increased while the other will be decreased. Without loss of generality, we assume that $s_{i}\left(t\right)<s_{j}\left(t\right)$ and the respective updates are described below.
  (5) $$\left\{\begin{array}{c} s_{i}\left(t+1\right)=s_{i}\left(t\right)+\delta ,\\ s_{j}\left(t+1\right)=s_{j}\left(t\right)-\delta \end{array}\right.$$

In addition, since support degrees cannot lie outside the interval [0,1], we apply the function $\prod_{\left[0,1\right]}$ below to limit the results obtained from Equations (2)–(5).

(6) $$\prod_{\left[0,1\right]}\left(x\right)=\left\{\begin{array}{cc} 1, & \;\mathrm{if}\;x>1\\ x, & \;\mathrm{if}\;0\leq x\leq 1\\ 0, & \;\mathrm{if}\;x<0 \end{array}\right.$$

For example, Equation (5) will turn into the following in our implementation.

(7) $$\left\{\begin{array}{c} s_{i}\left(t+1\right)=\prod_{\left[0,1\right]}\left(s_{i}\left(t\right)+\delta \right),\\ s_{j}\left(t+1\right)=\prod_{\left[0,1\right]}\left(s_{j}\left(t\right)-\delta \right) \end{array}\right.$$

**Proposition 4.**  *Suppose $R={\langle S\left(0\right),S\left(1\right),\cdots ,S\left(T\right)\rangle }_{M}$ is an observed evolution process that follows our POE model. If there exist $1\leq t^{*}\leq T$, s.t. $S(t^{*})$ is a positive (resp. negative, neutral) profile, then S(t) is also a positive (resp. negative, neutral) profile for $t^{*}<t\leq T$.*

**Proof.**  We simply prove the case for positive profiles and the other two are similar. In order to prove that S(t) is positive for $t^{*}<t\leq T$, we simply need to prove that $S(t^{*}+1)$ is also positive.

Without loss of generality, we assume that two agents, namely $a_{j}$ and $a_{k}$, are picked for interactions upon $S(t^{*})$. According to Definition 3, $s_{i}\left(t^{*}\right)>0.5$ for 1≤i≤N; thus $s_{j}\left(t^{*}\right)>0.5$ and $s_{k}\left(t^{*}\right)>0.5$. According to Equation (3), $s_{j}\left(t^{*}+1\right)=s_{j}\left(t^{*}\right)+\delta >0.5$ since δ>0. Similarly, $s_{k}\left(t^{*}+1\right)>0.5$. On the other hand, for any 1≤l≤N, s.t. l≠j and l≠k, $s_{l}\left(t^{*}+1\right)=s_{l}\left(t^{*}\right)>0.5$, so $s_{i}\left(t^{*}+1\right)>0.5$ for 1≤i≤N, which in turn confirms that $S(t^{*}+1)$ is a positive profile.    □

## 4. Simulations with POE Model

We visualized the properties of our model through Matlab simulations. To be specific, we demonstrated the effects of three parameters including (1) the *support degree change* δ, (2) the distribution of their initial *SDP* $\langle s_{1}\left(0\right),\cdots ,s_{N}\left(0\right)\rangle$ as well as (3) the group size *N*.

For each of the three parameters above, we evaluated how they influence the speed of convergence. So given a model with all parameters specified, we use $t^{*}$ to represent the average number of iterations needed to achieve convergence (see [50] for more details). Moreover, in order to observe convergence in a convenient way, we used an additional parameter $t_{\max}$ which means the number of iterations we perform in a particular run.

### 4.1. Comparing Different Values of Support Degree Change δ

For the simulations in this subsection, *N* and $t_{\max}$ were set to 200 and 10,000, respectively. To evaluate the impacts of δ, we tested each value in E(0.05,0.02,0.49) for this parameter. For each such value, we conducted simulations 500 times and obtained the $t^{*}$ value over these runs.

**Figure 1:**
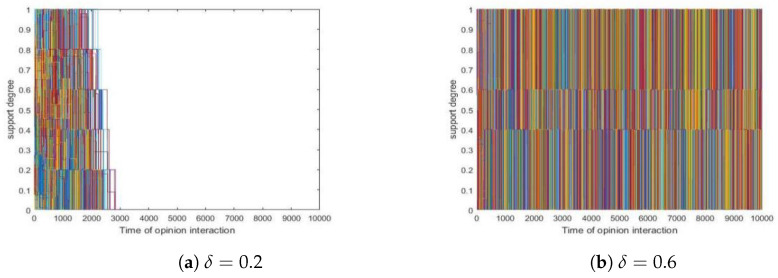
The average $t^{*}$ values for different δ values. (**a**) δ=0.2. (**b**) δ=0.6. Other parameters: N=200, $t_{\max}=10,000$, $s_{i}\left(0\right)∼U\left[0,1\right]$ where 1≤i≤200.

Since simulations showed that different δ values present similar trends concerning convergence, we took two runs as examples in which δ was set to 0.2 and 0.6, respectively, and we present them in Figure 1 here. Among all simulations, we found that those models with δ<0.5 quickly converge (form a consensus) while those with δ>0.5 failed to do so within $t_{\max}$ iterations, as is vividly shown in the two sub-figures of Figure 1.

**Figure 2:**
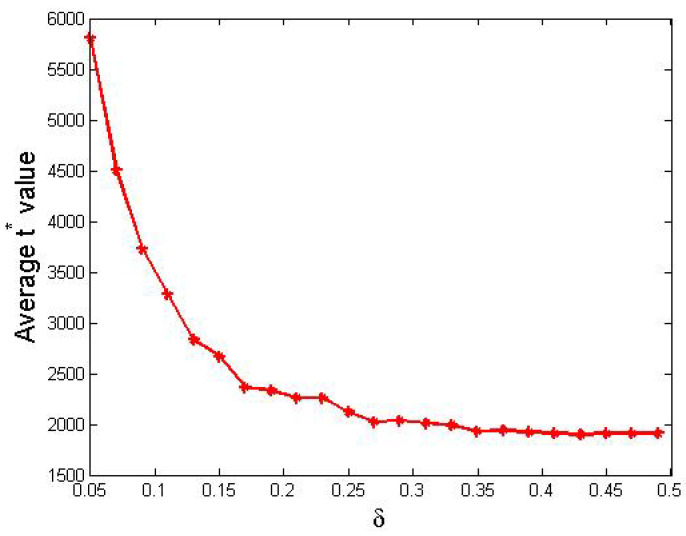
The average $t^{*}$ values for different δ values. Other parameters: N=200, $t_{\max}=10,000$, $s_{i}\left(0\right)∼U\left[0,1\right]$ where 1≤i≤200.

Furthermore, we present $t^{*}$ values with respect to different δ values in Figure 2.

From Figure 2, we obtained the following observations.

1. In general, the value of $t^{*}$ clearly decreased as δ increased from 0.05 to 0.49.
2. The decreasing trend of $t^{*}$ wrt. δ was sharp in the first half where δ ranged from 0.05 to 0.25 but became smooth in the second half, where δ is greater than 0.25.

Now, we analyze the performances visualized in Figure 2. When δ is small, agents can only update their support degree in small steps, so a great number of steps are needed to achieve consensus. In contrast, when δ is relatively big, a small amount steps are in need. On the other hand, when δ>0.5, agents’ support degrees update too fiercely so that no consensus was observed within $t_{\max}$ iterations.

**Remark 1.**  *In practice, given a society, when an average agent is reluctant to change its idea, it will take longer for the society to form a consensus. On the other hand, if an average agent is too open-minded, its opinion may keep changing and thus a consensus is difficult to achieve.*

### 4.2. Comparing Different Distributions of Initial SDP

We considered different distributions of agents’ initial *SDP* $\langle s_{1}\left(0\right),\cdots ,s_{N}\left(0\right)\rangle$, and evaluated their impacts on the result and speed of convergence. To be specific, we conducted two lists of simulations.

1. The former list evaluated the influences of different proportions of opinions, where the support degree distribution is uniform in both the positive and the negative groups.
2. The latter list simulated those initial *SDP*s that follow the beta and normal distribution, compared to those that follow the uniform distribution.

#### 4.2.1. The Effects of Different Proportions of Opinions

Given a fixed number of *N* agents, we partitioned them into two groups, those who support or oppose an issue. In this sense, we use $N_{p}$ and $N_{n}$ to denote the number of agents in these groups, respectively, and obviously, $N=N_{p}+N_{n}$.

**Figure 3:**
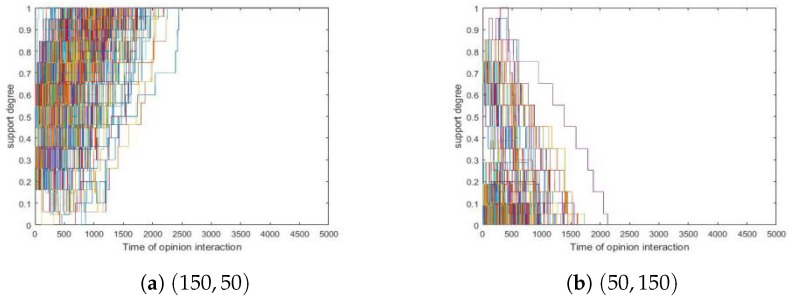
The effects for different $\left(N_{p},N_{n}\right)$ values. δ=0.1, N=200, $t_{\max}=10,000$. (**a**) (150,50). $s_{i}\left(0\right)∼U\left[0.5,1\right]$ where 1≤i≤150. $s_{151}\left(0\right),\cdots ,s_{200}\left(0\right)∼U\left[0,0.5\right]$. (**b**) Parameter settings are analogous.

Since simulations showed that different $\left(N_{p},N_{n}\right)$ values present similar results about convergence, we took two runs as examples in which $\left(N_{p},N_{n}\right)$ were set to (150,50) and (50,150), respectively, and we presented them in Figure 3 here.

From Figure 3, we obtained the following.

1. Figure 3a showed simulations where $N_{p}$ and $N_{n}$ were 150 and 50, respectively, and this simulation formed a consensus where all agents were positive.
2. Figure 3b showed similar situations where $N_{p}$ and $N_{n}$ were 50 and 150 and finally, all agents became negative.

From Figure 3, we conjectured that $N_{p}>N_{n}$ leads to a consensus where all agents are positive, while $N_{p}<N_{n}$ causes the opposite. To verify this claim, we conducted four groups of simulations where $\left(N_{p},N_{n}\right)$ were set to (180,20), (120,80), (90,110) and (30,170), respectively. In each group, we conducted 500 simulations and in the end, we made observations that fitted this conjecture.

**Remark 2.**  *In practice, if everyone is open-minded to some extent, then their meeting is likely to form a consensus that is consistent with majority votes, provided a sufficient number of interactions.*

#### 4.2.2. Evaluating Beta and Normal Distributions of Initial SDP

**Figure 4:**
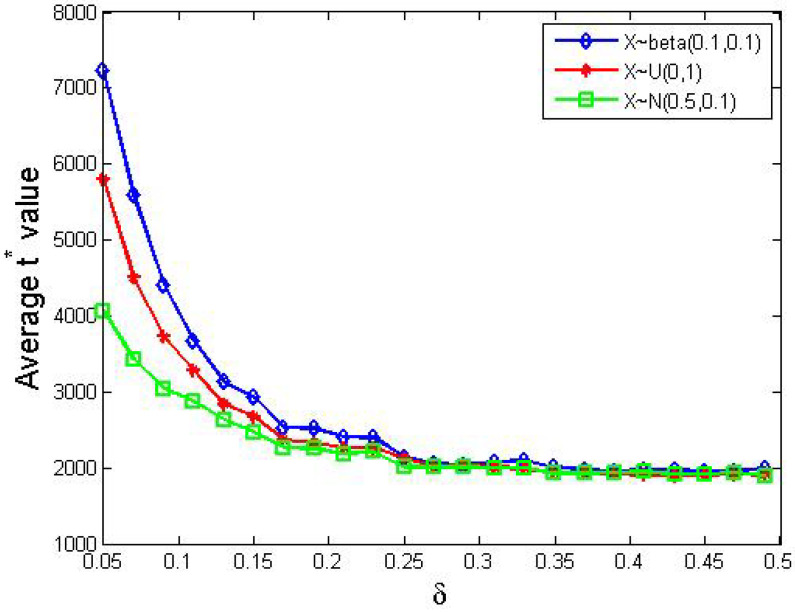
The influence of distributions of initial SDPs. Other parameters: N=200, $t_{max}=10,000$; the blue, red and green curves correspond to the beta, uniform and normal distribution, respectively.

In reality, agents’ support degrees can be concentrated to some extent. To be specific, there are two types of interesting distributions: (1) distributions where the majority are quite indifferent between supporting or opposing an issue, and (2) those where the majority have polarized support degrees. Rigorously, we think that the beta and the normal distributions are interesting because they reflect the two situations above. Hence, we redid the simulations in Section 4.1, but replaced the uniform distribution there with beta(0.1,0.1) and N(0.5,0.1), respectively. Then we visualized the results of these 3 distributions and placed their curves together in Figure 4.

From Figure 4, we obtained the following.

1. Obviously, the three curves shared similar trends with the one in Figure 2.
2. The beta distribution took the longest to form a consensus, while the normal distribution took the shortest time when δ is relatively small.

Further simulations showed that no consensus would be reached when δ>0.5. All in all, this figure illustrated that more concentrated distributions lead to sooner consensus among agents.

**Remark 3.**  *In reality, when most agents have initial support degrees that are similar, such agents can easily persuade others to accept their ideas. In contrast, if there exists a considerable amount of agents with polarized support degrees, it will take longer to persuade them to accept intermediate ideas.*

### 4.3. The Effects of Group Size

**Figure 5:**
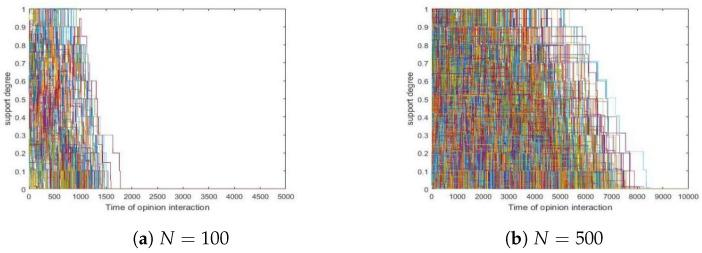
Influence of *N*. (**a**) N=100. (**b**) N=500. Other parameters: δ=0.1, $t_{\max}^{\left(a\right)}=5,000$, $t_{\max}^{\left(b\right)}=10,000$, the initial support degrees are uniformly and randomly selected from [0,1].

**Figure 6:**
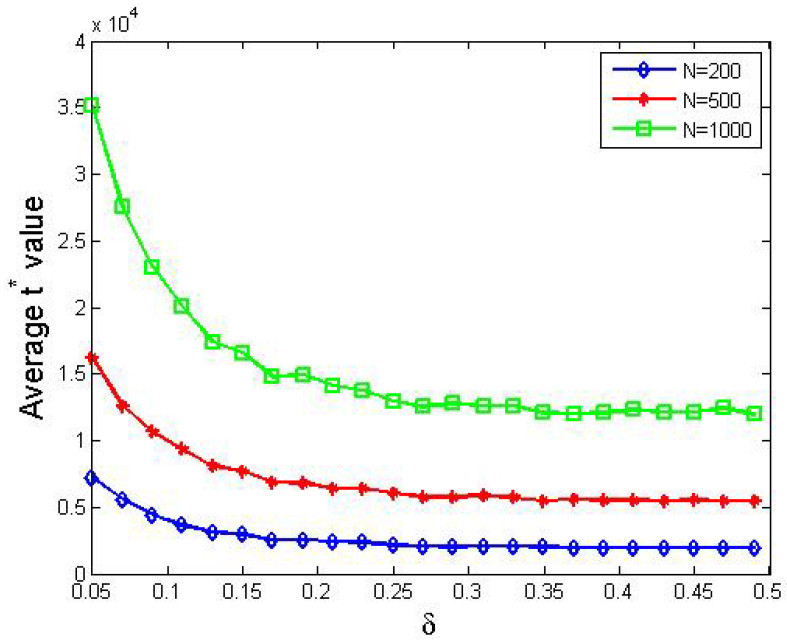
The average $t^{*}$ values for different δ values. Other parameters: the initial support degree is uniformly and randomly selected from [0,1]. The blue, red and green curves correspond to parameter combinations, namely (1) N=200, $t_{max}=10,000$; (2) N=500, $t_{max}=50,000$; and (3) N=1,000, $t_{max}=50,000$, respectively.

We redid the simulations in Section 4.1, but replaced the value of *N* with 100, 200, 500 and 1000, respectively. Since simulations showed that different *N* values present similar trends concerning convergence, we took two runs as examples in which *N* was set to 100 and 500, respectively, and we presented them in Figure 5 here. Furthermore, we visualized the results of 200, 500 and 1000 agents and placed their curves together in Figure 6.

In Figure 5, we found the following.

1. Both runs formed a consensus.
2. Larger groups of agents led to later consensus.

From Figure 6, we obtained the following.

1. Obviously, the three curves shared similar trends with the one in Figure 2, which indicated that whether their support degrees converge does not depend on the number of agents involved.
2. The situation in Figure 5 also occurred in the three cases here.

**Remark 4.**  *In a society where communications are primitive, to be specific, in each time stamp, only two agents are allowed to interact with each other, the time needed to form a consensus is proportional to the number of agents.*

### 4.4. Non-Uniform SDCs in A Group

In previous simulations, all agents have the same SDC. Alternatively, any two agents update their support degree with the same increase or decrease. In this subsection, we considered agents that could have individual SDCs, so we redid the simulation in Section 4.1, but replaced the uniform SDC among agents with individual ones.

**Figure 7:**
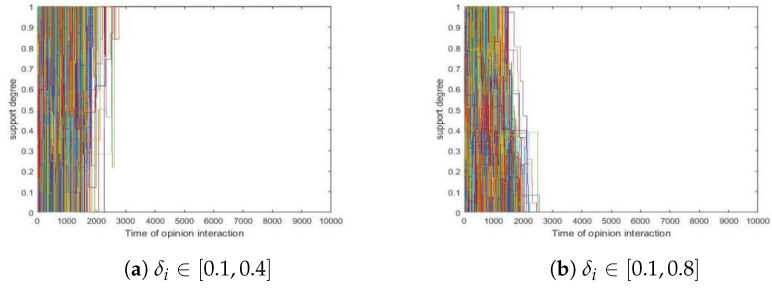
Non-uniform SDCs in a group. (**a**) $\delta_{i}\in \left[0.1,0.4\right]$. (**b**) $\delta_{i}\in \left[0.1,0.8\right]$. Other parameters: N=200, $t_{max}=10,000$, $s_{i}\left(0\right)∼U\left[0,1\right]$ where 1≤i≤200.

We conducted two simulations in which individual SDCs follow uniform distributions over [0.1,0.4] and [0.1,0.8], respectively. In what follows, we use $\delta_{i}$ to denote the $a_{i}$’s SDC. Since simulations showed that different runs present similar trends concerning convergence, we took two of them as examples in which $\delta_{i}∼U\left[0.1,0.4\right]$ and $\delta_{i}∼U\left[0.1,0.8\right]$, respectively, where 1≤i≤200, and we presented them in Figure 7 here.

In Figure 7, we found the following.

1. Both initial distributions formed a consensus in the end.
2. In Figure 7b, even though there were a significant proportion of agents whose SDCs were greater than 0.5, a consensus was formed eventually.

**Figure 8:**
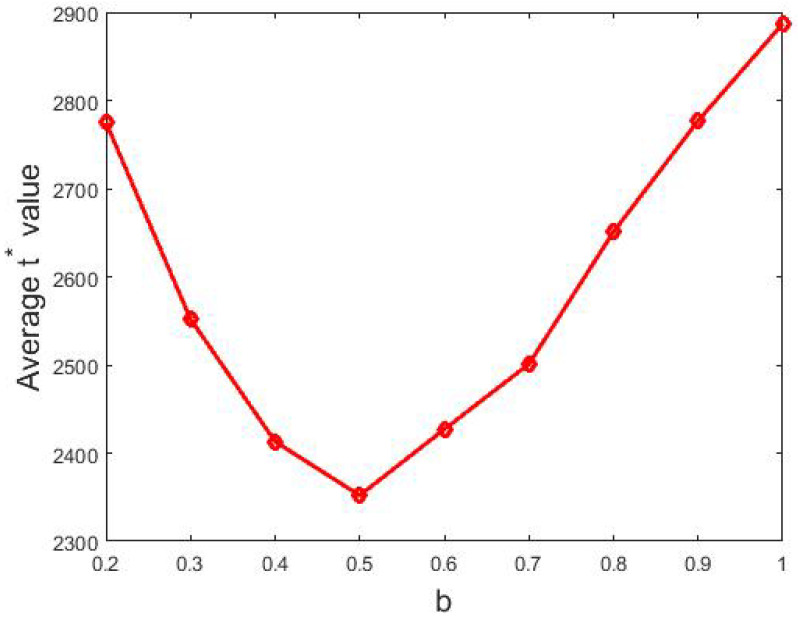
The relation between the average $t^{*}$ value and the right endpoint *b*. Other parameters: N=200, $t_{max}=10,000$, $s_{i}\left(0\right)∼U\left[0,1\right]$ where 1≤i≤200.

Furthermore, we considered other intervals namely [0.1,b] where b∈{0.2,0.3,⋯,1}. and each of them was tested 500 times. Then, we visualized the relation between *b* and $t^{*}$ in Figure 8 below.

From Figure 8, we obtained the following.

1. Even though there could be a significant proportion of agents who were more open-minded, i.e., they updated their support degrees considerably, a consensus was still reached.
2. The $b-t^{*}$ curve presented a decrease when b<0.5 but then showed an increase until *b* reached 1. This indicated that larger SDC values generated an earlier consensus when they were smaller than 0.5. Moreover, it revealed that more open-minded agents with SDC value greater than 0.5 produced a later consensus.

Now, we analyze this phenomenon.

1. Since there was a fair proportion of agents with SDC values less than 0.5, they constituted a core that served as a foundation for opinion evolution. Such a core persuaded those open-minded agents with SDC values greater than 0.5 to eventually agree with the opinions of the core.
2. As to the speed of convergence, when δ was small, it took longer to form a consensus which coincided with the mechanism beneath Figure 2. However, when b≥0.5, agents’ support degrees update quite fiercely, so it is not easy to reach a consensus, which was why more time was needed to reach a consensus.

**Remark 5.**  *As mentioned above, if agents in a society are too open-minded, they will hardly form a consensus. However, if there exist plenty of agents who are willing to update their support degrees in small steps, they will constitute a core and this core will gradually persuade those open-minded ones and finally turn them into their like-minded companions.*

## 5. Three Mechanisms for Polarization

Group polarization is a hot topec in recent research of opinion dynamics [50,51,52,53,54,55,56,57]. In this section, we propose three extensions of our POE model which served for polarization.

### 5.1. Communications Limited by Support Degree Differences

In reality, there can be communication barriers between agents whose support degrees differ too much. More concretely, if two agents have different opinions, their communication cannot occur unless their support degrees are close to some extent, i.e., the difference between their support degrees is smaller than a certain specified confidence threshold.

Based on our POE model above, we introduce a bounded confidence threshold ϵ, where 0≤ϵ≤1, which permits or prohibits communications between agents with different opinions. Actually, our intuition for this is as follows.

1. Agents with the same opinion communicate with each other effectively.
2. Only when two agents meet with different opinions, do we exert a threshold.

Formally, in Cases ②, ③ and ⑤ in Table 1, agents’ support degrees update only when $|s_{i}\left(t\right)-s_{j}\left(t\right)|\leq \epsilon$ for some confident specified threshold ϵ. Combining these rules and the ones in Equations (2)–(4) in Section 3, we have a novel model, named ϵ-POE model, for communications that are limited by support degree differences. Notice that such a model will degenerate to the POE model when ϵ=1. In addition, since support degrees cannot lie outside the interval [0,1], we implement this model in the same way as Equation (7).

**Figure 9:**
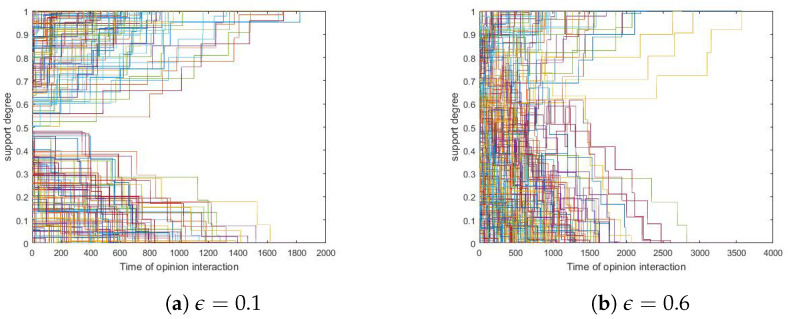
The influence of ϵ. (**a**) ϵ=0.1. (**b**) ϵ=0.6. Other parameters: δ=0.1, N=200, $t_{max}=10,000$, $s_{i}\left(0\right)∼U\left[0,1\right]$ where 1≤i≤200.

Since simulations showed that different ϵ values present similar trends in polarization, we took two particular runs as examples in which ϵ were set to 0.1 and 0.6, respectively, and we presented them in Figure 9 here.

Figure 9 shows that both the ϵ-POE modes (with ϵ=0.1 and ϵ=0.6, respectively) polarized.

**Figure 10:**
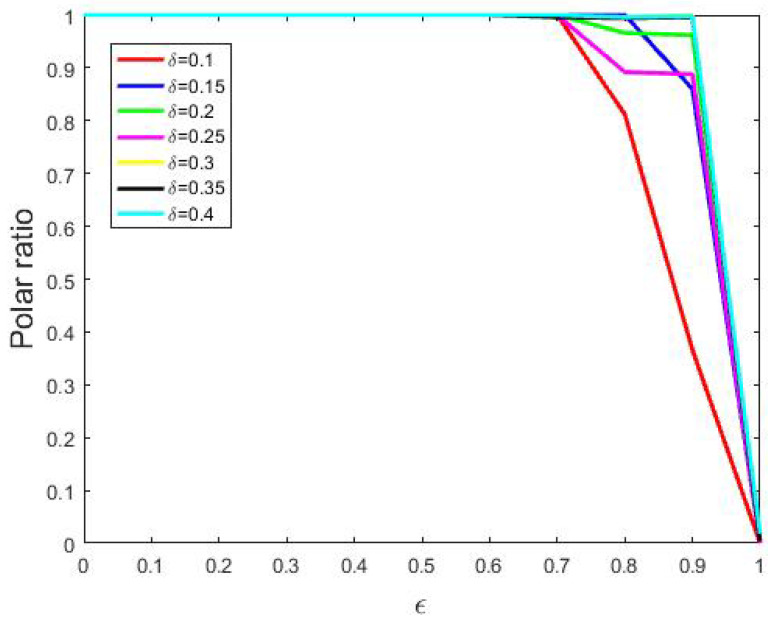
The effects of bounded confidence. Other parameters: N=200, $t_{max}=$ 10,000, $s_{i}\left(0\right)∼U\left[0,1\right]$ where 1≤i≤200.

Next, we tested all combinations of 〈δ,ϵ〉∈E(0.1,0.05,0.4)×E(0,0.1,1) and ran simulations 500 times with each of them. In this sense, we defined *polar ratio* as the proportion of runs that achieved polarization. The relation between polar ratios and bounded confidence is presented in Figure 10, in which each curve corresponds to a specific δ value.

From Figure 10, we found that larger bounded confidence led to smaller polar ratios, i.e., small bounded confidence tended to polarize. The reason may be that smaller bounded confidence results in less willingness to update one’s opinions.

**Remark 6.**  *More trust between agents with different opinions leads to less polarization.*

Actually, we have a proposition below which shows that in our ϵ-POE mode, once an SDP becomes 1-gap, it will preserve this property till the end of our observation.

**Proposition 5.**  *Suppose that M is an ϵ-POE model, and $R={\langle S\left(0\right),S\left(1\right),\cdots ,S\left(T\right)\rangle }_{M}$ is an observed evolution process. If there exists $1\leq t^{*}\leq T$, s.t. $s_{i}\left(t^{*}\right)\in \left\{0,1\right\}$ for all 1≤i≤N, then $S\left(t^{*}\right)=S\left(t^{*}+1\right)=\cdots =S\left(T\right)$.*

### 5.2. Polarization through More Effective Interaction with Like-Minded Agents

In reality, like-minded agents tend to communicate somewhat effectively. In this sense, like-minded agents cause more updates compared to those with different opinions. To distinguish between the effects resulting from like-minded agents and that from opposite-minded ones, we introduce an extra parameter 0≤c≤1 for perturbation which helps depict such prejudice. More specifically, we believe that support degree changes between like-minded agents should be enlarged by a factor of 1+c, while those between different-minded agents should be shrunk by a factor of 1−c. Hence, when like-minded agents meet, the update should be δ(1+c), which is greater than that in previous sections. Analogously, when opposite-minded agents meet, the update should be δ(1−c). If c=0, this model degenerates to the POE model above. By considering these issues, we have a model below which depicts such a situation.

1. The most trivial case is that both agents are neutral and the update rules are just the same as before, i.e., no updates are needed.
2. If both agents are positive (resp. negative) at time *t*, their confidence will be strengthened and thus their support degrees will be increased (resp. decreased) by δ(1+c), as is shown in Equations (8) and (9).
  (8) $$\left\{\begin{array}{c} s_{i}\left(t+1\right)=s_{i}\left(t\right)+\delta \left(1+c\right),\\ s_{j}\left(t+1\right)=s_{j}\left(t\right)+\delta \left(1+c\right) \end{array}\right.$$
  (9) $$\left\{\begin{array}{c} s_{i}\left(t+1\right)=s_{i}\left(t\right)-\delta \left(1+c\right),\\ s_{j}\left(t+1\right)=s_{j}\left(t\right)-\delta \left(1+c\right) \end{array}\right.$$
3. If two agents with different opinions meet each other, one support degree will be increased while the other will be decreased. So their support degrees will still get close, even though by a smaller step in this case. Without loss of generality, we assume that $s_{i}\left(t\right)<s_{j}\left(t\right)$ and the respective updates are described below.
  (10) $$\left\{\begin{array}{c} s_{i}\left(t+1\right)=s_{i}\left(t\right)+\delta \left(1-c\right),\\ s_{j}\left(t+1\right)=s_{j}\left(t\right)-\delta \left(1-c\right) \end{array}\right.$$

In addition, since support degrees cannot lie outside the interval [0,1], like in previous situations, we implement this model in the same way as Equation (7).

**Figure 11:**
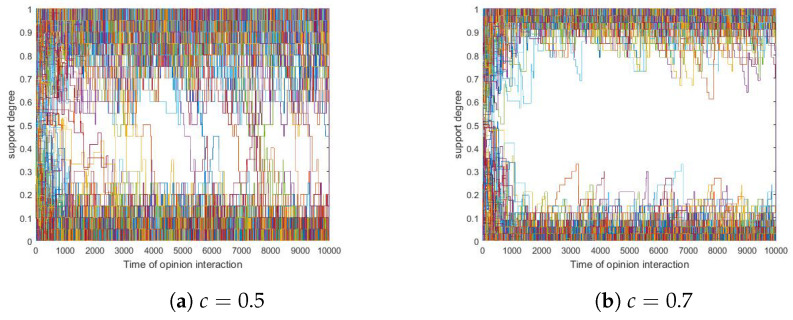
The influence of *c*. (**a**) c=0.5. (**b**) c=0.7. Other parameters: δ=0.1, N=200, $t_{max}=10,000$, $s_{i}\left(0\right)∼U\left[0,1\right]$ where 1≤i≤200.

Since simulations showed that different *c* values present similar trends, we took two specific runs as examples in which *c* were set to 0.5 and 0.7, respectively, and we presented them in Figure 11 here.

In Figure 11, neither consensus nor $\tau_{0}$-polarization was observed with $\tau_{0}\geq 0.8$. Yet detailed observations showed that $\tau_{0}$-polarization was observed with $\tau_{0}>0.6$. To better depict this phenomenon, we propose Definition 8 below.

**Definition 8.**  *(dynamic polar) We counted the number of people in the interval [0,r], and [1−r,1], which are separately denoted by η, and μ, if $|\frac{\eta }{N}-\frac{\mu }{N}|\leq p$, and $\frac{\eta }{N}+\frac{\mu }{N}>q$, where 0<r≤1, 0≤p≤1, and 0≤q≤1, then we say that a dynamic polar among the agents is reached at time t, which concerns r, p and q.*

**Figure 12:**
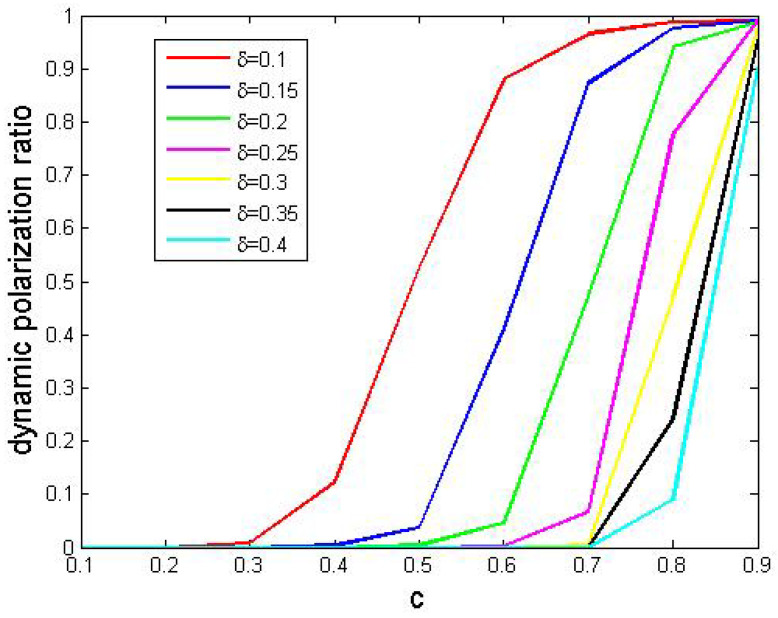
The effects of the perturbation parameter *c*. Other parameters: N=200, $t_{max}=10,000$, $s_{i}\left(0\right)$∼U[0,1] where 1≤i≤200.

Below, in each simulation, we set r=0.4, p=0.2 and q=0.95. Then, we considered all combinations of δ∈E(0.1,0.05,0.4) and c∈E(0.1,0.1,0.9), and we ran simulations 500 times with each combination. Finally, we reported dynamic polarization ratios in Figure 12, in which each curve corresponded to a δ value.

In Figure 12, we found that bigger δ values produced smaller dynamic polarization ratios, which indicated that small δ values tend to cause polarization. Moreover, we noticed that larger perturbation values led to greater dynamic polarization ratios.

Now, we analyze the performances. With bigger δ, agents’ support degrees fluctuate sharply so that it is difficult to realize dynamic polarization. On the other hand, if agents communicate much more effectively with like-minded ones compared to opposite-minded ones, their support degrees rarely come close to the average level among them.

**Remark 7.**  *First, we considered a conservative society in which the majority are stubborn, i.e., they are little willing to change their support degrees. The less their willingness is, the more likely they are to form a dynamic polar.*

*Second, we considered a society where individual agents have obvious prejudice, i.e., they update their support degrees more strongly with like-minded companions. The greater their prejudice is, the more probable it is that they will reach a dynamic polar.*

### 5.3. Polarization through the Higher Chance of Interaction with Like-Minded Agents

Inspired by the Barnum Effect [58], we considered a case where agents desire to interact with like-minded companions. Such interactions can positively reinforce one’s own beliefs. However, in the POE model, we assume that any two agents have equal opportunities for interactions. To be specific, each agent is chosen for communications with a probability about $\frac{1}{N}$, where *N* is the number of agents. In this sense, they have a 50/50 chance of being like-minded.

Given a particular agent $a_{i}$, we use $\rho (a_{i})$ to denote the proportion of agents that share the same opinion with agent $a_{i}$, so the proportion of agents that have different opinions is $1-\rho (a_{i})$. Then we introduce a bias parameter 0≤b≤1, which helps increase the probability that an agent meets like-minded companions. More specifically in our setting, if an agent $a_{i}$ supports or opposes an issue, it will meet like-minded companions at a probability of $\min\{\rho \left(a_{i}\right)+b,1\}$ while it meets other agents at a probability of $1-\min\{\rho \left(a_{i}\right)+b,1\}$. However, if agent $a_{i}$ feels neutral about that issue, it will meet any other agent with equal probability. Notice that such a model will degenerate to the POE model above when b=0.

Our model here is the same as the POE model before with a single exception that we pick agents for communications by Algorithm 1.
**Algorithm 1:** PickAgentPair.
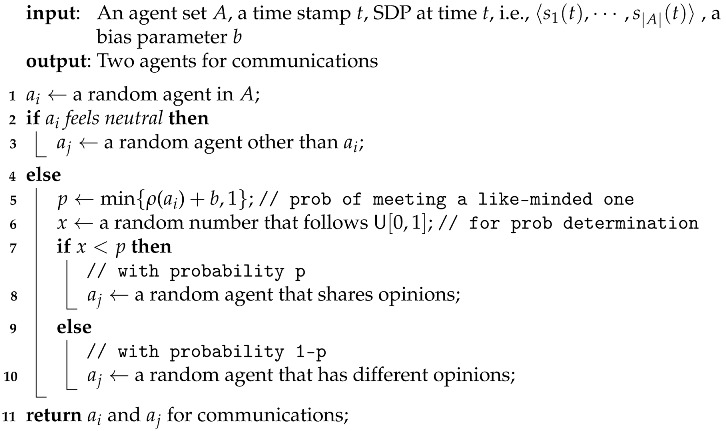


In addition, since support degrees cannot lie outside the interval [0,1], like in previous situations, we excluded unreasonable values in the same way as Equation (7).

**Figure 13:**
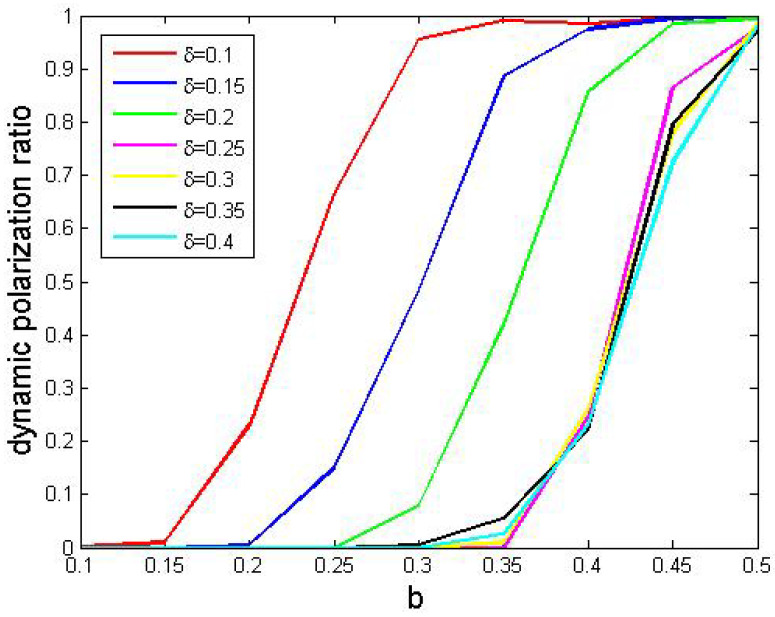
The effects of perturbation parameters. Other parameters: N=200, $t_{max}=10,000$, $s_{i}\left(0\right)∼U\left[0,1\right]$ where 1≤i≤200.

Below, in each simulation, we set r=0.4, p=0.2 and q=0.95. Then, we considered all combinations of δ∈E(0.1,0.05,0.4) and b∈E(0.1,0.05,0.5) and then tested their effects. We found that the results were similar to those presented in Figure 11; therefore, we also used Definition 8 to depict such phenomena. We ran simulations with all combinations of parameters, 500 times for each. Finally, we reported dynamic polarization ratios in Figure 13, in which each curve corresponded to a δ value.

In Figure 13, we found the following.

1. Obviously, those curves shared similar trends with the one in Figure 12.
2. More perturbation resulted in higher dynamic polarization ratios.

Based on such observations, we conjectured that more concentration on like-minded companions could cause more dynamic polarization.

**Remark 8.**  *In a society where agents tend to communicate with like-minded companions, their support degrees will probably be increased by each other. Hence, the society may be divided into several subgroups each of which shares close support degrees. In other words, these agents will likely form a dynamic polar.*

## 6. Conclusions

In this paper, we proposed a hybrid opinion dynamic model based on progressive opinion evolution with a discrete component, namely agents’ opinions, as well as a continuous one, namely support degrees. It has two distinguishing features as follows. (1) When agents meet with someone with the same opinions, their opinions will be strengthened; to be specific, their support degrees could increase or decrease simultaneously. (2) Agents may not be able to achieve an agreement (to have the same support degree) in a single interaction. Moreover, we proposed several extensions to this POE model which serve as mechanisms of opinion polarization. To be specific, the first extension introduced a further component, namely confident threshold, that limited communications between different-minded agents. The second extension considered prejudice on different-minded agents, more specifically, like-minded agents produced greater updates while different-minded agents generated smaller ones. The third brought about more opportunities for communication between like-minded agents.

We conducted a series of simulations to explore the behaviour of our models. In particular, we evaluated the impacts of several components and model parameters on the results and speeds of convergence. The results of these simulations show that our models reflected some aspects of social reality and thus simulated some social phenomena.

For future works, we will explore models with dictatorships. Moreover, it will be interesting to investigate an agent society with interactions that involve more than two agents.

## Data Availability

Not applicable.
